# The relationship between amniocentesis results and the existence of secondary symptoms in the anomaly scan in mothers referring to Sabzevar Perinatology Clinic between 2016 and 2019

**DOI:** 10.61622/rbgo/2026rbgo65

**Published:** 2026-09-23

**Authors:** Sana Mokhlesi, Masoumeh Sharifzadeh, Mohammad Nemat Shahi, Mina Ghalenovi, Mozhdeh Navi Nezhad, Mitra Eftekhariyazdi

**Affiliations:** 1 Sabzevar University of Medical Sciences Faculty of Medicine Sabzevar Iran Faculty of Medicine, Sabzevar University of Medical Sciences, Sabzevar, Iran.; 2 Sabzevar University of Medical Sciences Mobini Hospital Sabzevar Iran Mobini Hospital, Sabzevar University of Medical Sciences, Sabzevar, Iran.; 3 Sabzevar University of Medical Sciences Faculty of Medicine Department of Anesthesia Sabzevar Iran Department of Anesthesia, Faculty of Medicine, Sabzevar University of Medical Sciences, Sabzevar, Iran.; 4 Sabzevar University of Medical Sciences Faculty of Nursing and Midwifery Department of Midwifery Sabzevar Iran Department of Midwifery, Faculty of Nursing and Midwifery, Sabzevar University of Medical Sciences, Sabzevar, Iran.; 5 Sabzevar University of Medical Sciences Mobini Hospital Sabzevar Iran Mobini Hospital, Sabzevar University of Medical Sciences, Sabzevar, Iran.; 6 Sabzevar University of Medical Sciences Faculty of Medicine Department of Obstetrics and Gynecology Sabzevar Iran epartment of Obstetrics and Gynecology, Faculty of Medicine, Sabzevar University of Medical Sciences, Sabzevar, Iran.

**Keywords:** Amniocentesis, Ultrassonography, Genetics screening, Down syndrome, Pregnancy trimester, second

## Abstract

**Objective:**

The importance of observing some secondary symptoms found in the ultrasound scan for anomalies is still unknown. This study aims to investigate the chromosomal results of amniocentesis in cases of detecting secondary symptoms in the ultrasound scan within the second trimester of pregnancy.

**Methods:**

This descriptive-analytical study was conducted between the years 2016 and 2019 on 310 pregnant women whose gestational age was between17+6 to19+6 weeks and showed secondary symptoms in the ultrasound scan within the second trimester. In high-risk cases, after determining the final risk, an amniocentesis sample was prepared and the results of the fetal karyotype were examined. Demographic characteristics, pregnancy information, ultrasound results, and genetic tests were extracted from mothers’ files.

**Results:**

Focusing on the ultrasound scan,235 individuals showed only one secondary symptom,59 individuals came with two secondary symptoms, and four individuals had three or more secondary symptoms. Moreover, 10 individuals had other structural abnormalities. Having the amniocentesis sampled from all the individuals with secondary symptoms, only 11(3.5%) were genetically abnormal, including one person with trisomy 18(0.3%), three people with trisomy 21(1%), one person with Turner syndrome (0.3%), one person with Klinefelter syndrome (0.3%), and the rest (five individuals) had translocation, isochromosome, and extra chromosome (1.6%). No significant relationship was found between the number of secondary symptoms in the ultrasound scan for anomalies and the chromosomal abnormalities.(P=0.9)

**Conclusion:**

Nasal bone hypoplasia and intracardiac echogenic focus are the most common secondary symptoms. Down syndrome is the most common trisomy in the studied population.

## Introduction

Screening by ultrasound in the second trimester has significantly advanced in order to identify fetal abnormalities. It is now possible to detect even minor structural abnormalities. However, some sonographic findings are considered minor or clinically insignificant. These symptoms are known as "secondary symptoms" and are mostly seen in fetuses with congenital or chromosomal abnormalities.^(1)^ Ultrasound findings in fetuses with Down syndrome include both structural and non-structural markers. Nevertheless, other aneuploidies such as trisomy 13, trisomy 18, monosomy X and triploidy can also be detected by ultrasound. Fetuses with aneuploidy are checked through invasive tests such as placental villi sampling or amniocentesis.^(2)^ However, these procedures are associated with an increased risk of miscarriage and other potential complications including musculoskeletal deformities. Therefore, many women prefer to avoid having such tests.^(1)^

Although some secondary symptoms can be seen in normal fetuses, due to the increased incidence of chromosomal and congenital abnormalities, the relationship between some of these markers with fetal intrauterine death, premature birth, fetal growth restriction, and congenital infection in the euploid embryos are considered important secondary symptoms.^(3,4)^ It is estimated that secondary symptoms are present in 11% to 17% of normal fetuses.^(5)^

These markers are not the same in different races and may appear independently of major structural abnormalities.^(6)^ Usually, the secondary symptoms studied are thickened nuchal fold, mild pyelectasis, nasal bone hypoplasia, echogenic intracardiac focus, hyperechoic bowel, and short femurs and humerus. However, in some studies, in addition to the above cases, single arterial umbilical cord, ventriculomegaly, and choroid plexus cyst are also considered secondary symptoms.^(7,8)^

Since no similar research has been conducted to detect the level of aneuploidy in the high-risk group in terms of the secondary symptoms of ultrasound in Sabzevar, considering the difference in its incidence and prevalence in different regions as well as the importance of determining the normal range of soft markers in the Iranian population, this study aimed to investigate the results of amniocentesis in the cases of secondary symptoms in the ultrasound scan for anomalies in the second trimester in mothers referred to Sabzevar Perinatology Clinic from 2016 to 2019.

## Methods

Using convenience sampling, this descriptive-analytical study was undertaken on all pregnant mothers who referred to the perinatology clinic of Sabzevar University of Medical Sciences between 2016 and 2019, who had sub-markers of abnormal ultrasound scans and were placed in the high-risk group after determining the final risk. Inclusion criteria include the willingness of parents to perform amniocentesis despite any type of secondary symptom, a gestational age of less than 20 weeks, being in the high-risk group after determining the risk in the ultrasound scan, abnormality of the scan with the secondary symptom, the presence of a secondary symptom in the ultrasound, the absence of undertaking serum screenings, and the availability of complete information in the clinical file. Exclusion criteria included multiple pregnancy, unwillingness to cooperate, inability to follow up due to referring to other centers outside the city the sampling carried out, withdrawal of mothers due to fear of amniocentesis complications, not affording the costs of amniocentesis and genetic testing, and the absence of growth for the sample.

Data collection was carried out through a checklist made by the researcher after obtaining mothers’ consent to use their information. The checklist contains personal and pregnancy information of the mother such as: age, parity, gravid, gestational age based on LMP (Last Menstrual Period) and NT, underlying diseases of the mother and problems in previous pregnancies, presence of secondary symptoms in ultrasound, and also determined risks in other screenings.

To state the procedure, those women whose ultrasound scan for anomalies was undertaken within weeks 17+6 to 19+6 and reported secondary symptoms confirmed by a perinatologist were included in the study if the risk of each secondary symptom and the risk of serum screening put them in the high-risk group. Amniocentesis was performed after advising of the complications and costs and obtaining written consent. Before amniocentesis, for all pregnant mothers, ultrasound was performed to confirm the viability of the fetus, determining the location of placenta and amniotic fluid volume, and estimating the best point for inserting the needle. Afterwards, the abdomen was swabbed with an antiseptic solution and sampled using the spinal needle No. 20, considering transabdominal ultrasound. To prevent contamination with maternal cells, 1-2 milliliters of the sample was discarded and about 1 milliliter per week of pregnancy (average 20 milliliters) of amniotic fluid was taken by two 10-milliliter syringes and sent to the laboratory for examination. The color of the amniotic fluid (clear, green, brown, bloody) was recorded in the file. The tests performed on the sent samples according to the cost and satisfaction of the participants included karyotype, QFPCR (Quantitative Fluorescent Polymerase Chain Reaction), MLPA (Multiplex Ligation-dependent Probe Amplification). Finally, having received the results from the laboratory, they were recorded in the clinical file. The researcher entered the genetic test results in the relevant questionnaire, using the files in the clinic.

The data were analyzed by SPSS version 26 using descriptive tests (mean, standard deviation, frequency, and frequency percentage) and analytical tests (Chi-square and Fissure Exact test). P<0.05 was considered significant.

This study was conducted after obtaining the code of ethics IR.MEDSAB.REC.1398.27 from the Vice-Chancellor for Research at Sabzevar University of Medical Sciences. In order to use the clinical files, the coordination was made with the management of the special clinic and the head of the perinatology department, and the sampling permit was registered. Moreover, the written consent of the couples was obtained for sampling.

## Results

310 individuals whose average age was 30.15 ± 7.15 years participated in this study. In terms of BMI (Body Mass Index), 35.5% of the participants had a normal rate, 2.4% had a lower-than-normal rate, and 11% had a BMI at the overweight or obese level. In terms of education, most people in the group had a diploma or lower certificates (74.5%), 1.6% were illiterate, and the rest had academic education (less than 1.0%). The average number of pregnancies was 2.40±1.31, deliveries 1.10±1.03, and abortions 0.29±0.56 (Table 1). In addition, 11 individuals (3.5%) mentioned the history of chromosomal disorders in their family (Table 1).

**Table 1:** Demographic characteristics and pregnancy information of the individuals participated in the research

Items	Minimum	Maximum	Mean	Standard Deviation
Age (year)	14	46	30.15	7.15
Gravida	1	8	2.40	1.31
Parity	0	5	1.10	1.03
Number of abortions	0	2	0.29	0.56
**Items**	**n(%)**			
Occupation				
Housewife	288(92.9)			
Employed	22(7.1)			
Education				
Illiterate	5(1.6)			
Diploma and lower	231(74.5)			
Academic education	74(23.9)			
History of chromosomal disorders				
Yes	11(3.5)			
No	229(96.5)			

Two-hundred thirty-five individuals (75.8%) had one soft marker in the ultrasound scan of anomalies. 59 individuals (19%) showed two secondary symptoms and four people (1.3%) had three or more markers. In 12 individuals, other abnormal cases were observed such as choroid plexus cyst, ventriculomegaly, absence of stomach visibility, omphalocele and clinodactyly, hydrocephalus, aortic coarctation, single arterial umbilical cord, AVSD, and hydronephrosis (Figure 1).

**Figure 1:**
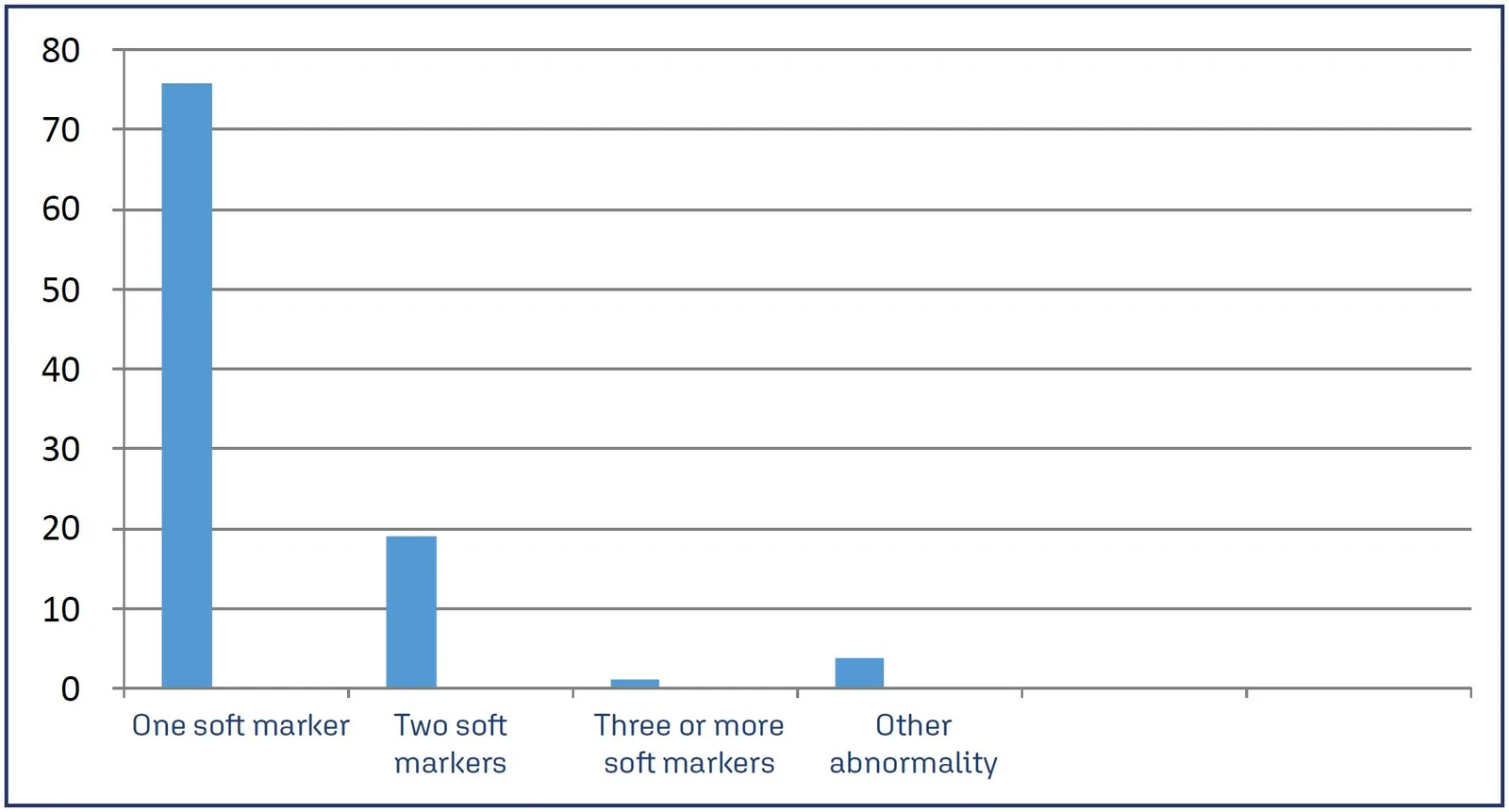
The number of abnormal markers observed in the ultrasound scan for anomalies

In terms of prevalence of secondary symptoms observed in abnormal ultrasound, as shown in table 2, the highest and lowest frequencies were hypoplasia of the nasal bone (71.6%) and shortness of humerus and femur (2.3%), respectively.

**Table 2:** Prevalence of secondary symptoms observed in the ultrasound scan for anomalies

Secondary symptoms	n(%)
Increasing the thickness of the fold on the back of the neck of the fetus	12(3.9)
Hypoplasia of the nasal bone	222(71.6)
Intracardiac echogenic focus	49(15.8)
Echogenic intestine	24(7.7)
Pyelectasis	35(11.3)
Shortness of the humerus and femur	7(2.3)
Other abnormalities	28(9)

In terms of the type of test performed on amniocentesis samples, on 209(68.4%) samples, only QF-PCR test, on 31.3%, karyotype test, and on the rest (0.3%) MLPA test was performed. Among 310 abnormal ultrasound scans that were analyzed for karyotype, only 11(3.5%) were genetically abnormal. Trisomy 18, trisomy 21, Turner syndrome, Klein filter syndrome and other abnormalities such as translocation, isochromosome and extra chromosome were observed (Table 3).

**Table 3:** Frequency distribution of genetic results obtained from amniocentesis samples

Genetic results		n(%)
Normal		299(96.5)
Abnormal	Trisomy 21	3(1)
	Trisomy 18	1(0.3)
	Turner	1(0.3)
	Klinefelter	1(0.3)
	Translocation, and	3(0.96)
	Isochromosome	2(0.64)
	Extra chromosome	0(0)
Total		310(100)

In terms of the relationship between identified chromosomal abnormalities and the number of secondary symptoms seen in the ultrasound anomaly scan, the results of the chi-square test did not show a statistically significant relationship between the number of markers and chromosomal abnormalities (P=0.9). Statistically speaking, this means that there is no relationship between the number of soft markers and the abnormality of chromosomes (Table 4). The aim of the current study was to investigate the relationship between the presence of secondary signs in second trimester ultrasound anomaly scan in mothers and amniocentesis results, regardless of the serum screening results (Tables 5 and 6).

**Table 4:** The relationship between chromosomal abnormalities and secondary symptoms in the ultrasound scan for anomalies

Abnormalities	Normal chromosome n(%)	Abnormal chromosome n(%)	p-value
One secondary symptom	226(72.9)	9(2.9)	
Two secondary symptoms	57(18.3)	2(0.6)	0.9
Three secondary symptoms	4(1.3)	0(0)	
Other abnormalities	12(3.9)	0(0)	

**Table 5:** Results of first trimester serum screening

Failure to perform screening (%)	High risk (chance of risk greater than 1 in 250) (%)	Moderate risk (probability of risk between 1 in 250 and 1 in 1500) (%)	Low risk (risk probability less than 1 in 1500) (%)	First trimester screening
118(38.1)	38(12.3)	50(16.1)	104(33.5)	Trisomy 21
118(38.1)	0	0	192(61.9)	Trisomy 18
118(38.1)	0	0	192(61.9)	Trisomy 13

**Table 6:** Results of second trimester serum screening

Failure to perform screening (%)	High risk (%)	Low risk (%)	Second trimester screening
154(49.7)	88(28.4)	68(21.9)	Trisomy 21 (cutoff point 250)
154(49.7)	2(0.6)	154(49.7)	Trisomy 18 (cutoff point 100)
154(49.7)	0	156(50.3)	Trisomy 13 (cutoff point 100)

## Discussion

This study examined the association between secondary ultrasound markers and chromosomal abnormalities detected via amniocentesis in a population of pregnant women. Among 310 participants with abnormal second-trimester anomaly scans, nasal bone hypoplasia was the most frequently observed soft marker. Only 3.5% of these cases were confirmed to have chromosomal abnormalities through genetic testing, with trisomy 21 being the most prevalent. In a study by Hurt (2016), the prevalence of intracardiac echogenic foci was 43.7 cases in 1000 pregnancies.^(9)^ Masihi et al. (2021) undertook a study in the city of Ahwaz and identified 148 (9.4%) fetuses with choroid plexus cysts, 4 (0.13%) fetuses with mega cisterna magna and one fetus with thickened nuchal fold and mild ventriculomegaly at the same time.^(6)^ In the study by Shi et al., nasal bone hypoplasia was observed as the most common secondary symptom (41.9%).^(10)^ This difference could be due to the fact that in this study, all the examined cases were positive in terms of trisomy 21, which explains why the frequency of the secondary symptom was higher. In addition, the type of soft markers examined was different. However, hypoplasia in the anomaly scan of the second trimester is considered one of the important secondary symptoms.

In the current study, only 11 cases (3.5%) in 310 abnormal ultrasound scans, which were sampled for genetic testing, were genetically abnormal. In the study by Hu et al. (2021), the overall prevalence of chromosomal abnormalities in fetuses with secondary symptoms was 2.3% (107 in 2466), of which 2.4% had chromosomal numerical abnormalities, 6.48% had pathogenic copies, and 2.11% was associated with possible pathogenic copies.^(11)^ In the study by Hildebrand et al. (2021), 13% of all abnormalities in the first trimester scan and 29% in the second trimester scan were identified. In this study, it was stated that the advantages of having an ultrasound scan in the second trimester are the detection of abnormalities of the heart, urinary system, CNS, and chromosomal abnormalities.^(12)^

Cai et al. (2021) identified 46 cases (4.06%) of chromosomal abnormalities among 1131 fetuses with secondary symptoms.^(13)^

In the study by Masihi et al. (2021), among 118 cases with mild pyelectasis, only one case had a major heart disorder and two cases had miscarriage. The phenotype of both pregnancies with the thickened nuchal fold led to miscarriage. Two in eight cases of isolated mild ventriculomegaly had major structural abnormalities. Two cases of abortion were reported. The researchers stated that mothers who have fetuses with choroid plexus cysts or mild pyelectasis irrelevant to other structural abnormalities should be reassured that pregnancy outcomes are generally favorable.^(6)^ In the study by Hu et al., the occurrence of numerical chromosomal abnormalities in the presence of several secondary symptoms (5.5% vs. 1.5%; P = 0.001) and the thickened nuchal fold (8.3% vs. 7.1%; P = 0.024) was significantly higher.^(11)^ Hurt et al. in their study observed the association between the isolated intestinal echogenicity and an increased risk of congenital malformations (RR=4.54) and premature delivery (RR=2.30). Isolated pyelectasis was associated with the risk of congenital malformations (RR=3.82). Multiple markers increase the risk of congenital anomalies (RR=0.5) and premature birth (RR=3.38).^(9)^ Ahman et al. stated that the presence of soft markers shows a positive probability for Down syndrome (probability ratio = 1.7), but this relationship was statistically significant only in the group of soft markers (isolated, multiple or in combination with other abnormalities).

Invasive diagnostic testing increased 24-fold when isolated soft markers were observed, almost irrespective of women's age. Soft markers must be mentioned when information on second trimester ultrasound is formulated and all units performing fetal ultrasound examinations must have established routines concerning information management when soft markers are identified

Through a four to nine-year-old follow-up of babies born with soft markers identified by pregnancy ultrasound, a piece of research in 2019 by Tosun et al. showed that among 28 fetuses suffering from intracardiac echogenic foci, there were allergic asthma and epilepsy in two infants. Nine pregnancies with echogenic bowel were followed. Epilepsy was observed in one case. Allergic asthma was diagnosed in both UCPC and BCPC (Unilateral and Bilateral Choroid Plexus Cysts), while epilepsy and attention deficit hyperactivity disorder (ADHD) were diagnosed in two infants with BCPC. Given the existence of several soft markers, 12 infants were examined. Except for one case of allergic asthma, one case of hearing loss, and one case of ADHD, no pathology was observed in most cases. This study shows favorable long-term outcomes of infants with isolated or multiple soft markers without any aneuploidy and may provide insight into this debated issue.^(4)^

Differences between studies may be due to the differences in the type of soft markers investigated or different tests performed on amniocentesis samples. In our study, QF-PCR was performed on most of the samples, and yet, some chromosomal abnormalities may not have been identified. In the current study, nasal bone hypoplasia and intracardiac echogenic focus were the most common secondary symptoms. The Down syndrome, also, was the most common trisomy diagnosed. Likewise, a study by Zhou et al. stated that the presence of several soft markers in ultrasound increases the risk of chromosomal abnormalities. Absence of nasal bone or hypoplastic nose was the most important clinical indicator alone or in combination with other ultrasound soft markers.^(14)^ However, Hu et al. reported that various types of chromosomal aberrations and pathogenic copies may co-exist with soft markers, especially multiple soft markers. It is necessary to undertake genetic investigations when observing these symptoms in ultrasound.^(11)^ Ahman et al. reported that the probability ratio for Down syndrome was significant for each marker (isolated, multiple, or with abnormalities).^(1)^ While soft markers such as nasal bone hypoplasia are frequently detected, their predictive value for chromosomal abnormalities remains limited when isolated. Nevertheless, a structured approach combining clinical judgment, risk stratification, and genetic testing is essential for informed prenatal care.

Of strengths of this study, it is important to mention that the presence of secondary symptoms in the ultrasound performed in other centers in the clinic was examined and confirmed again by a perinatologist. All diagnostic procedures were performed by a specialist and an assistant, and all samples were sent to a laboratory. However, there were also limitations, including the failure of all individuals referring to the perinatology clinic to undergo an anomaly scan ultrasound, the failure of some mothers to refer to the perinatology clinic after the detection of soft markers and the failure to respond to follow-ups, the high cost of amniocentesis and screening tests, and the low number of cases of complete karyotyping and chromosome microarray. Future studies should incorporate comprehensive genetic testing, such as chromosomal microarray, to improve detection of submicroscopic abnormalities. Moreover, research should aim to define normative ranges for soft markers within specific populations, including the Iranian demographic, to enhance screening specificity and reduce unnecessary invasive procedures.

The results of this study can be a starting point for studies in regard to determining the normal range of soft markers in the Iranian population.

## Conclusion

This study showed that hypoplasia of the nasal bone was the most common soft marker observed in the ultrasound scan for anomalies. Similarly, the most common chromosomal abnormality identified in the amniocentesis samples was trisomy 21. There was no statistically significant relationship between the number of secondary symptoms in the ultrasound scan for anomalies and chromosomal abnormality in amniocentesis.

## Data Availability

The research data are described in the article presented.

## References

[1] Åhman A, Axelsson O, Maras G, Rubertsson C, Sarkadi A, Lindgren P (2014). Ultrasonographic fetal soft markers in a low-risk population: prevalence, association with trisomies and invasive tests. Acta Obstet Gynecol Scand.

[2] Ali MK, Shazly SA, Ali AH, Abdelbadee AY, Abbas AM (2012). Ultrasonographic soft markers of aneuploidy in second trimester fetuses. Middle East Fertil Soc J.

[3] Kim MS, Kang S, Cho HY (2018). Clinical significance of sonographic soft markers: A review. J Genet Med.

[4] Tosun M, Kurtoglu Ozdes E, Malatyalioglu E, Yavuz E, Celik H, Bildircin FD (2019). Long-term outcome of fetuses with soft marker and without genetic or structural abnormality. J Obstet Gynecol India.

[5] Kaplan R, Adams S (2018). Incidental fetal ultrasound findings: interpretation and management. J Midwifery Womens Health.

[6] Masihi S, Barati M, Karimi Moghaddam E, Rezazadeh A, Ronaghi F (2021). Outcome of fetuses with soft markers: results of 3016 cases in Ahvaz city. Med J Islam Repub Iran.

[7] Rebarber A, Levey KA, Funai E, Monda S, Paidas M (2004). An ethnic predilection for fetal echogenic intracardiac focus identified during targeted midtrimester ultrasound examination: A retrospective review. BMC Pregnancy Childbirth.

[8] Hung KL, Liao HT (2002). Neonatal choroid plexus cysts and early childhood developmental outcome. J Formos Med Assoc.

[9] Hurt L, Wright M, Dunstan F, Thomas S, Brook F, Morris S (2016). Prevalence of defined ultrasound findings of unknown significance at the second trimester fetal anomaly scan and their association with adverse pregnancy outcomes: the Welsh study of mothers and babies population-based cohort. Prenat Diagn.

[10] Shi XM, Fang Q, Chen BJ, Xie HN, Xie YJ, Chen JH (2013). [Investigation of ultrasound markers in screening fetal trisomy 21]. Zhonghua Fu Chan Ke Za Zhi.

[11] Hu T, Tian T, Zhang Z, Wang J, Hu R, Xiao L (2021). Prenatal chromosomal microarray analysis in 2466 fetuses with ultrasonographic soft markers: a prospective cohort study. American journal of obstetrics and gynecology.

[12] Hildebrand E, Selbing A, Blomberg M (2010). Comparison of first and second trimester ultrasound screening for fetal anomalies in the southeast region of Sweden. Acta Obstet Gynecol Scand.

[13] Cai M, Lin N, Chen X, Fu M, Guo N, Xu L (2021). Evaluation of chromosomal abnormalities and copy number variations in fetuses with ultrasonic soft markers. BMC Med Genomics.

[14] Zhou Y, Wu S, Han J, Zhen L, Yang X, Li R (2023). Prenatal diagnosis of ultrasound soft markers in a single medical center of mainland China. Mol Cytogenet.

